# Trial Protocol: Randomised controlled trial of the effects of very low calorie diet, modest dietary restriction, and sequential behavioural programme on hunger, urges to smoke, abstinence and weight gain in overweight smokers stopping smoking

**DOI:** 10.1186/1745-6215-11-94

**Published:** 2010-10-07

**Authors:** Deborah Lycett, Peter Hajek, Paul Aveyard

**Affiliations:** 1 UKCTCS, Primary Care Clinical Sciences, University of Birmingham, UK; 2 Tobacco Dependence Research Centre, Barts and The London School of Medicine and Dentistry, Queen Mary University of London, UK

## Abstract

**Background:**

Weight gain accompanies smoking cessation, but dieting during quitting is controversial as hunger may increase urges to smoke. This is a feasibility trial for the investigation of a very low calorie diet (VLCD), individual modest energy restriction, and usual advice on hunger, ketosis, urges to smoke, abstinence and weight gain in overweight smokers trying to quit.

**Methods:**

This is a 3 armed, unblinded, randomized controlled trial in overweight (BMI > 25 kg/m^2^), daily smokers (CO > 10 ppm); with at least 30 participants in each group. Each group receives identical behavioural support and NRT patches (25 mg(8 weeks),15 mg(2 weeks),10 mg(2 weeks)). The VLCD group receive a 429-559 kcal/day liquid formula beginning 1 week before quitting and continuing for 4 weeks afterwards. The modest energy restricted group (termed individual dietary and activity planning(IDAP)) engage in goal-setting and receive an energy prescription based on individual basal metabolic rate(BMR) aiming for daily reduction of 600 kcal. The control group receive usual dietary advice that accompanies smoking cessation i.e. avoiding feeling hungry but eating healthy snacks. After this, the VLCD participants receive IDAP to provide support for changing eating habits in the longer term; the IDAP group continues receiving this support. The control group receive IDAP 8 weeks after quitting. This allows us to compare IDAP following a successful quit attempt with dieting concurrently during quitting. It also aims to prevent attrition in the unblinded, control group by meeting their need for weight management. Follow-up occurs at 6 and 12 months.

Outcome measures include participant acceptability, measured qualitatively by semi-structured interviewing and quantitatively by recruitment and attrition rates. Feasibility of running the trial within primary care is measured by interview and questionnaire of the treatment providers. Adherence to the VLCD is verified by the presence of urinary ketones measured weekly. Daily urges to smoke, hunger and withdrawal are measured using the Mood and Physical Symptoms Scale-Combined (MPSS-C) and a Hunger Craving Score (HCS). 24 hour, 7 day point prevalence and 4-week prolonged abstinence (Russell Standard) is confirmed by CO < 10 ppm. Weight, waist and hip circumference and percentage body fat are measured at each visit.

**Trial Registration:**

Current controlled trials ISRCTN83865809

## Background

Weight gain is a well known consequence of smoking cessation [1,2]. It may deter smokers from attempting to quit [3] and offsets some advantages of giving up smoking. Smoking cessation-related weight gain partly explains the finding that the incidence of type II diabetes is increased by 50-100% in the years after cessation [4,5] and there is a 30% increased risk of hypertension [6] compared to continuing smoking. The improvement in lung function of quitters decreased by 38% in men and 17% in women as a consequence of weight gain [7].

An ideal intervention to prevent weight gain would begin at the time of quitting, because weight gain is most rapid initially, and then the rate of gain slows [8]. However, initial weight management is controversial. The main reason quit attempts fail is due to quitters succumbing to their cravings to smoke. Evidence suggests that hunger increases urges to smoke (smokers smoke more when hungry)[9,10] and people who gain most weight are more likely to succeed in quitting smoking[11]. These observations led to attempts to reduce cigarette cravings by using glucose tablets, which was shown to be effective[12]. This suggests that avoiding hunger and feeding cigarette cravings with food may enhance smoking abstinence. Therefore current smoking cessation advice is to avoid hunger and not to diet during a quit attempt, although healthy food choices are often advocated. However the hypothesis that dieting while quitting increases hunger and thereby increases urges to smoke in the early phases of a quit attempt has not been tested in free living conditions. DeMiST is designed to test this hypothesis and also builds on the results of a Cochrane review[13], which investigated weight gain prevention during smoking cessation. The reviewers reported that general dietary education to reduce energy intake through eating a low fat, healthy diet did not prevent weight gain compared to standard smoking cessation behavioural support. Furthermore, there was a statistically significant reduction in abstinence at 12 months (Table 1). An individually tailored dietary plan to reduce energy intake; with regular monitoring and adaptation of individual goals, reduced weight gain at 6 and 12 months; without a statistically significant reduction in abstinence rates (Table 1). Intermittent use of a very low calorie diet (VLCD) provided the greatest effect on preventing weight gain at end of treatment, but the effect was no longer statistically significant at 12 months and was lower than the individual plan. However use of the VLCD showed a statistically significant increased abstinence rate to almost double that of controls at end of treatment and long-term follow up (Table 1).

**Table 1:** Effects of dietary interventions on weight change and abstinence during smoking cessation.

Intervention compared to standard smoking cessation	Mean difference in weight change (Kg [95%CI])		Abstinence (RR [95%CI])	
	
	End of treatment	At 12 months	End of treatment	At 12 months
General lifestyle and calorie reducing dietary advice	-0.04 [-0.57, 0.50]	-0.21 [-2.28, 1.86]	0.90 [0.76, 1.06]	0.66 [0.48, 0.90]

Individually tailored dietary and lifestyle advice	-1.05 [-2.01, -0.09]	-2.58 [-5.11, -0.05]	1.11 [0.84, 1.46]	0.79 [0.47, 1.33]

VLCD compared to general calorie reducing dietary advice	-3.70 [-4.82, -2.58]	-1.30 [-3.49, 0.89]	1.40 [1.07, 1.85]	1.73 [1.10, 2.73]

The effect of the VLCD on improving abstinence is unexpected. One hypothesis is that the VLCD induced ketosis, which actually suppressed hunger [14] and therefore reduced urges to smoke. This hypothesis is supported by data supplied by Danielsson (personal communication), which showed a statistically significant reduction of urges to smoke and a smaller increase in appetite during the weeks on the VLCD diet. There was a 50% reduction in urges to smoke after 1 week on the VLCD diet compared to a 27% reduction after 1 week in the control group (P < 0.0001). There was a 4-fold increase in hunger in the control group after 1 week compared to only a 50% increase in hunger in the VLCD group (p < 0.0003).

However the intermittent use of the VLCD in this study might have concealed the full potential a VLCD to reduce nicotine withdrawal symptoms. Swinging in and out of a ketotic state is likely to have its own bearing on mood and hunger regardless of nicotine withdrawal. This study has also been criticised for lacking generalisibilty into a health service setting, for only recruiting women and only recruiting those who had previously failed in quit attempts because of weight concerns.

DeMiST includes daily cigarette smokers regardless of gender or previous quit attempts. Its primary aim is to investigate the feasibility of running dietary interventions as part of the National Health Service (NHS) stop smoking service. Its secondary aim is to investigate the association of hunger and urges to smoke. It will further test whether ketosis, induced by a VLCD, reduces urges to smoke through hunger suppression.

The effect of 3 dietary strategies on hunger and cigarette craving will be compared. One is the VLCD, the second is an individual dietary and activity plan (IDAP) which includes a modest energy restriction, using a low fat diet which meets the energy requirements of an individual's BMR. The third is a control condition where general healthy eating advice is provided but with an emphasis of avoiding hunger, this is typical of the current advice given in NHS stop smoking services.

We expect hunger to be greatest in the IDAP group where a moderate energy restriction creates a negative energy balance. Appetite is then increased in response to the usual physiological and neurological mechanisms that work to restore energy homeostasis [15]. In the control condition we are expecting hunger to be alleviated by eating freely.

The associations of hunger and craving scores within and between these groups will be investigated. These scores are recorded daily. The VLCD will begin one week before quit day to ensure participants are in ketosis before they quit (verified by the presence of urine ketones) and will continue, uninterrupted, for a further 4 weeks when nicotine withdrawal is at its peak. The IDAP will also begin one week before quit day to ensure that participants are in a state of hunger when they quit. The control group will be advised to eat as usual. Therefore at the time of quitting we expect to see the maximal differences in hunger scores between the 3 groups.

The greatest criticism of VLCDs in current clinical practice is that they may not establish long term healthy eating habits, although a review by NICE reported that a 5% reduction in body weight is maintained over 2 years in those following a VLCD plan [16]. Our aim is to use the results of this trial to inform a study large enough and of long enough duration to assess long term effects on dietary change, weight, cardiovascular risk, lung health and smoking abstinence. For this reason we have designed DeMiST to be a small scale model of such a trial. Therefore DeMiST extends beyond the initial quitting phase into a second treatment stage where both the VLCD and the control group receive individually tailored dietary advice.

The reason for this in the VLCD group is to provide them with conventional support to establish long term healthy habits. The reason for this in the control group is two-fold. Firstly, because the control is unblinded we hope that by providing IDAP after quitting this group will not feel 'short changed' and abandon their quit attempt prematurely. Secondly we will be compare the long term effects from IDAP, which restricts energy intake at the time of quitting, with the 'control' group which restricts it after they have already quit. There is evidence to suggest that the latter may be more successful than the former, although this hasn't been tested with an adequately powered trial, [17] we plan to do so in our future trial.

## Methods

### Participants

Overweight (BMI > 25 Kg/m^2^) smokers listed on the databases of participating general practices are invited to take part by a letter from their General Practitioner (GP). All participants are from practices in Birmingham East and North Primary Care Trust (PCT) and Worcestershire PCT, in the UK. (See additional file 1 for substantial amendment to recruitment strategy).

Interested participants telephone the trial office at The University of Birmingham for further information; they provide verbal consent to initial telephone screening for eligibility. If considered eligible they are given an appointment with a trial nurse for full screening where they are invited to give informed consent for participation. The trial office sends the patient information sheet out so that it is received at least 24 hours before their first appointment. The clinics are run at local GP practices, daytime appointments are offered from 8.30 am until 7 pm.

#### Inclusion Criteria

• Aged over 18 years

• Daily cigarette smoker with an exhaled CO of at least 10 ppm at least 15 minutes after last smoking

• BMI of at least 25 kg/m^2^

• Willing to be randomised to any of the three arms and willing and able to comply with the intervention and all study procedures

We are recruiting only those with a BMI greater than or equal to 25 for the following reasons: 1) The rate of weight loss on a VLCD is approximately 2 kg/week so anyone entering the trial would need to be able to lose 10 kg. For example, a woman 1.64 m tall weighing 68 kg with a BMI of 25.2 could potentially lose 10 kg and reach a healthy BMI of 21.6 kg/m^2^. VLCDs have been shown to be as safe in those with a BMI > 25 kg/m^2 ^as in those with a BMI > 30 kg/m^2 ^[18]. 2) Research shows that those who are overweight or obese are likely to gain more weight than healthy weight smokers and so they are an appropriate target for weight gain prevention [19]. 3) The smoking population has a lower mean BMI than the non-smoking population so we could potentially struggle to recruit only those with a BMI >30 kg/m^2^.

#### Exclusion Criteria

• Any of the absolute or relative contraindications to VLCD use. These include situations in which rapid weight loss would be unsafe: pregnancy, breastfeeding, myocardial infarction/unstable angina/acute coronary syndrome in the past 6 months, cerebrovascular accident/transient ischaemic attack/stroke in the past 3 months, major surgery in the last 3 months, severe cardiac arrhythmias, severe hepatic impairment, severe renal impairment (i.e. GFR ≤ 29 mls/min), active carcinoma, untreated gallstones, past history of anorexia nervosa or bulimia nervosa, type 1 diabetes, those aged over 70 years with a BMI < 30. A large energy deficit alters metabolic rate and so anyone with unstable thyroid function is also excluded. Sudden weight loss may cause fainting or precipitate gout in those who are susceptible so those with regular blackouts or fainting, and untreated gout are also excluded. The VLCD formula is made from milk so is unsuitable for anyone with a milk allergy or intolerance.

• Uncontrolled hypertension and type 2 diabetes treated with medication. Although these are not contraindications to the VLCD they are excluded as adjusting the medications in these conditions would require specialist advice beyond the scope of the research nurses in this trial (diet controlled type 2 diabetics may be included).

• Those on oral anticoagulants, digoxin, phenytoin and lithium are excluded due to likelihood of increased drug absorption if on a VLCD. Specialist advice beyond the scope of our research nurses is needed to reduce and monitor these medications.

• Those on diuretics for diuresis are excluded as a VLCD can potentiate diuresis and increase the risk of hypokalaemia. Those on low dose diuretics for treatment of hypertension are not excluded as the risk is of hypokalaemic complications on these are low [20,21]. The effects of combining a VLCD with low dose thiazides has not been well studied and may be unpredictable therefore serum potassium will be monitored weekly and the VLCD discontinued if levels fall below 3.1 mmol/l. (Additional file 2 contains full list of excluded medications).

• Previous severe adverse reaction to nicotine patches (that which precludes further use).

• Active phaeocromocytoma as this combined with use of NRT may increase the risk of hypertension and tachycardia.

• Currently using smoking cessation medication i.e. nicotine replacement therapy (NRT), Vareniciline or Bupropion or medication (e.g. nortriptyline) that is known to help smokers quit.

• Those taking weight loss medication e.g. orlistat, sibutramine.

• Suspected abuse of alcohol or other drug as this might confound our measure of cravings.

• Use of any smokeless tobacco

• Currently participating in other therapeutic clinical trials.

#### Removal of Patients from Therapy

• If a contraindication to treatment becomes apparent.

• An adverse event occurs that makes it inadvisable to continue treatment.

• If serum potassium falls below 3.1 mmol/l in those taking thiazides, the VLCD will be discontinued.

• The person ceases to continue to quit smoking and wishes also to abandon the weight management programme. If a person fails to stop smoking they may continue with the dietary treatment intervention, likewise if a person abandons the dietary treatment they may continue with their quit attempt. Keeping participants like this in the trial will help us to explore the reasons why a treatment was abandoned. We will find out whether abandonment of one treatment ultimately leads to abandonment of both and how these participants have decided to tackle their smoking or weight.

### Interventions

#### Treatment Stage 1

**Figure 1:**
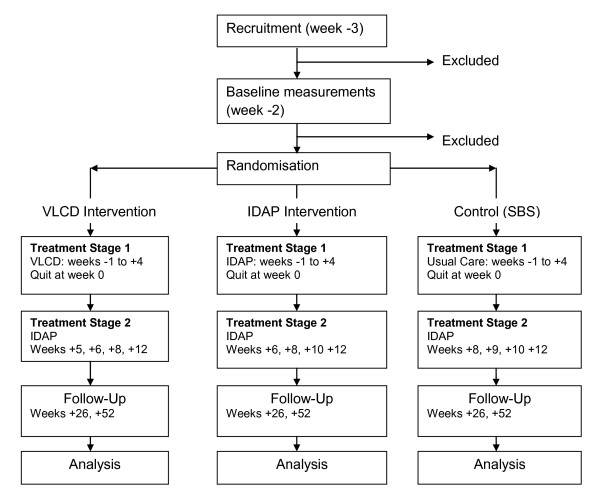
**Phases in DeMiST**.

This is for 8 weeks from -3 to + 4 (Figure 1). Week 0 is quit week, negatively numbered weeks are the weeks before quit day and positively numbered weeks are the weeks after quit week. Week -3 is baseline, three weeks before quit week. Participants are seen and briefed at baseline and a quit day is set for three weeks time. Dietary interventions commence one week prior to quit week (week -1). Participants are randomised into either the VLCD, IDAP or Control (SBS) arm as described below.

##### VLCD

The VLCD formula Lipotrim is provided for the study at cost price from Howard Foundation Research Ltd. It is purchased by participating Primary Care Trusts (PCTs) and provided free to participants. Participants take 2-3 shakes each day and drink a minimum of 4 pints of water, but not in excess of 4 litres. Black tea and black coffee may be drunk but no other food or drink can be consumed as any additional carbohydrate or citric acid may suppress ketone production. The female formula totals 425 kcal and the male formula totals 559 kcal, it contains all essential nutrients and complies with the EU codex standard for VLCDs [16]. This diet is begun 1 week before quitting, by which time the dieter should be in ketosis. They continue this diet during the first 4 weeks after quitting when nicotine withdrawal symptoms are at their worst. The rationale for this is that they will be comfortably in ketosis when they quit and when in acute nicotine withdrawal.

Every effort is made to get the participant to adhere to the VLCD by using behavioural change techniques as defined by Abraham and Michie [22]. These include an explanation of the role of ketones and the necessity for strict adherence in order to remain in a ketotic state. (Providing instruction and providing information on consequences of action are behavioural change techniques 2 and 8.) Ketosis is usually achieved by the third or fourth day on the diet and so participants are advised that they will feel better after this time. Encouragement (technique 6) and prompting of self-encouragement (technique 22) is used e.g. 'today was difficult but if I keep going I will feel better in 2 days', 'if I can stick this out, I can lose weight and stop smoking in time for my birthday'. Support to identify and overcome barriers to adherence (technique 5) is also given.

##### Individually Dietary and Activity Planning (IDAP)

This contains dietary, activity and behavioural elements. Assessment of current behaviours and food choices is made by using the Health Choice Index (HCI). This is a multiple choice questionnaire which asks the participant to score their food choices, weekly food frequency, eating behaviours and activity. From this, both the participant and the healthcare professional can identify areas for improvement and goals are then agreed. Typically three goals are given particular attention, one focussing on food choice e.g. I will buy skimmed milk instead of full fat milk; one on eating behaviour e.g. 'I will prepare a packed lunch the night before to take to work so that I don't miss lunch'. And one activity focussed goal e.g. 'I will park the car a block away from work and take a brisk walk at lunchtime so that I fit in 3 ten minute exercise breaks during my working day. Prompting specific goal setting is technique 10 in the behavioural change taxonomy. These goals are reviewed regularly and adjusted as necessary (behaviour change technique 11). Helping the participant to identify and overcome barriers to achieving their goals, discussing time management to incorporate these changes into their daily lives, helping them to plan relapse prevention strategies and general encouragement all form part of this intervention (behavioural techniques 5, 26, 23 and 6 respectively).

As well as identification of specific goals instruction is given (technique 8) for following a moderate calorie restricted diet plan tailored to the individual's energy requirements. This provides a structure to help consolidate the goals identified and ensure that the participant has the right tools to achieve a sufficient energy deficit for weight control.

The energy prescription used in previous studies to prevent weight gain on smoking cessation has varied. Pirie et al calculated daily energy requirement minus 150 to 300 kcal/day (which is considered to equate to metabolic slowing upon nicotine withdrawal, amount depends on number of cigarettes smoked [23]. Perkins et al used a 500 kcal deficit tailored to individual requirements [24]. Hall used a 500 kcal deficit tailored to individual requirements should weight increase by 1 kg [11]. Danielsson used a 1600 kcal diet [25].

We have decided to advise an approximate energy deficit of 600 kcal. This is recommended by NICE for weight loss of 0.5 kg/week [16]. This is sufficient to counter the mean rate of post-cessation weight gain and promote modest weight loss which is desirable for our population with a BMI > 25 kg/m^2^. Dietetic consensus considers a usual maximum energy prescription of 1800 kcal. Anecdotally advising above this appears to be ineffective at achieving weight loss, this may be due to a people misunderstanding the volumes of food recommended for larger portion sizes.

To calculate energy prescription in individuals we have assumed all participants will have a physical activity level (PAL) of 1.4, which is consistent with a sedentary occupation and leisure activities. The PAL is the factor by which BMR is multiplied to give an estimate of total energy expenditure. For simplicity in clinical practice we have written dietary plans that equate to energy intakes of 1200 kcal, 1500 kcal or 1800 kcal. An individual is allocated the closest energy prescription to their BMR (as calculated by the Tanita body composition analyser). Calculating total energy requirement from a PAL of 1.4 and BMRs of 1200 kcal, 1500 kcal and 1800 kcal, shows energy requirement is an increase of 500 kcal-700 kcal above BMR. This means we can approximately achieve the 600 kcal deficit by using BMR alone for the prescription of energy. The energy level advised is translated into the appropriate number of portions from each of the food groups: complex carbohydrates, fruit and vegetables, meat, fish and alternatives, dairy foods, sugar and fatty foods. The proportion of food coming from these food groups make up a healthy diet with 15-30% of energy from fat, 10-15% of energy from protein, 55-75% of energy from carbohydrate and 0-5% energy from alcohol. Therefore the diet is low in fat and alcohol, two things that studies have shown significantly increase in the diets of smokers when they quit [26-28]. The dietary plan is a 'pick and choose' format, where individuals choose from a list of items within different food groups at each meal. The food portions are defined in American cup sizes and participants are given measuring cups to measure out their portions.

##### Control (Step by Step (SBS))

The control group receives healthy eating advice when they mention weight concern. This is standard in smoking cessation interventions. Advice is given not to 'diet' but instead to avoid hunger by eating healthy, low fat foods. This is because it is thought that hunger may lead to urges to smoke, and may make relapse more likely. Tips for avoiding hunger include:

• Regular meals, including breakfast (which is often missed in smokers)

• Handy, healthy snacks e.g. fruit or chopped vegetables (carrot, celery sticks)

• Drink plenty of water, particularly before meals, this helps fill the stomach and satisfy 'oral cravings'.

There is evidence that glucose tablets satiate urges to smoke within minutes, so these can be used[12]. Avoidance of excess or total alcohol for the immediate post-cessation period is advised. This is to avoid lapsing due to disinhibition and the 'cue' which alcohol provides, rather than to avoid calories. Participants are advised to increase daily activity, e.g. including a 30 minute walk in their day. Advice in the control group is given as general instruction and general encouragement (behavioural techniques 6 and 8) but individual goal setting and energy prescription is not included.

#### Treatment Stage 2

This spans from week +4 to +12 and includes 4 visits (Figure 1). The same number of visits has been included in each of the intervention arms so that number of consultations with a healthcare professional is not a confounder.

##### VLCD

The VLCD is discontinued at week +4, participants are weaned back onto food. This weaning takes 1 week; there is a gradual reducing of the lipotrim formula which is replaced by low fat meals with a gradual increase of carbohydrate until healthy proportions are achieved. Gradual reintroduction of carbohydrate is necessary in this way to avoid rapid replenishing and storage of muscle glycogen, which could result in a rapid increase of body water weight (up to 5 kg over the week). At week +5 lifestyle goals are set and energy prescription is given as described in treatment stage 1 of IDAP. Behavioural support continues at reduced frequency to week +12. The reason for this second stage of treatment is to cultivate the development of long term healthy habits as explained in the background section of this paper.

##### Individually Tailored Dietary Planning (IDAP)

This behavioural support continues to week +12 with visits becoming less frequent.

##### Control (Step by Step (SBS))

Participants in the control arm, receive IDAP from week +8 to week +12. This allows them time to become an established quitter before they embark on weight control.

#### Smoking Cessation Interventions for all Patients

All participants have identical treatment to stop smoking. This is the treatment which is available on the NHS. It is withdrawal orientated such that behavioural strategies and nicotine replacement are given to relieve withdrawal symptoms. This has been shown to be an effective model for stopping smoking [29]. Individuals are seen over 7 consecutive weeks, weeks -3 and -2 prepare them for quitting, they quit at week 0, weeks +1, +2, +3 and +4 support them during the first 4 weeks after quitting when withdrawal symptoms are at their strongest. The sessions incorporate a variety of behavioural change techniques, pre-quit sessions prompt intent formation (technique 4) by setting a quit date, they prompt barrier identification (technique 5) and relapse prevention (technique 23) when participants identify times or places when it will be particular hard for them to resist smoking, they discuss a strategy to help them to deal with these circumstances; for example, changing routine so the usual smoking cues are removed. Quit week and the weeks that follow provide instruction (technique 8) in the use of nicotine replacement, review of and further planning of strategies (technique 11) to deal with withdrawal symptoms and general encouragement (technique 6). Self-talk (technique 22) is encouraged such that participants are asked to see and describe themselves as a 'non-smoker' and to frequently bring to mind the benefits of quitting that they are looking forward to.

##### Nicotine replacement therapy (NRT)

Nicorette 25 mg transdermal 16 hour patches are provided for 8 weeks followed by a 15 mg patch and a 10 mg patch each for 2 weeks. Patches are supplied free of charge by McNeil Products Ltd. Other forms of NRT, such as gum or nasal spray or a combination of NRT is not used to avoid the possibility that the amount of NRT taken would differ by arm. This could confound effects of hunger or ketosis on urges to smoke that we are trying to discover. We are using a nicotine patch as it delivers a consistent amount of nicotine to avoid this confounding. As no other NRT products can be used in combination to help with acute cravings we are using the 16 hour, 25 mg patch which has been shown to yield better abstinence than other available patches [30].

#### Training and supervision

Treatment is provided by trained practice nurses. Training is given by NHS stop smoking services (2 days), a research nurse and a general practitioner (GP) (1/2 day), and a registered dietitian (1.5 days). Nurses are given the clinical protocol to read before training sessions. Training includes explanation of dietetic and behavioural interventions and practicing the interventions on each other. The clinical protocol is clarified where needed and questions answered. The aim of the training is to equip the nurses so that, once they have completed it, they feel confident to deliver the interventions according to the clinical protocol.

After the training, 'hands on' supervision is available for the first few clinics. Immediate telephone access to the dietitian and GP is available for all clinical queries throughout the rest of the trial.

Medical history and any medication used or altered during the trial is monitored. Any significant changes in clinical condition of individual participants, as measured on a weekly basis, is discussed with the supervising GP and action taken, e.g. clinically significant fall in blood pressure in participants on the VLCD taking anti-hypertensives will require adjustment of anti-hypertensive medication. The participant's own GP is kept informed.

#### Fidelity checking and monitoring

Fidelity to the clinical protocol and record keeping is assessed and monitored against the clinical protocol by the principal investigator every few months; consultations are audiotaped for this purpose. Any deviations are recorded, discussed and corrected either immediately or at following clinics. The trial is potentially subject to audit by the appropriate regulatory authorities and therefore participants are asked to consent to allow their records to be viewed.

### Objectives

#### Primary objectives

To investigate the feasibility of running this three-armed dietary intervention as part of the NHS stop smoking services using primary care nurses,. This includes the feasibility of measuring and monitoring of physiological and biochemical risk factors for cardiovascular disease, diabetes and chronic obstructive pulmonary disease (COPD). It explores the acceptability of the interventions to participants.

#### Secondary objectives

To investigate whether smoking cessation advice and a nicotine patch in combination with: a very low calorie diet (VLCD), or individually tailored dietary planning (IDAP), or usual support affects urges to smoke, through hunger or ketosis in overweight smokers trying to quit.

#### Tertiary objectives

To investigate the extent to which changes in smoking status, diet and activity achieved during the treatment stages are maintained at the end of treatment and at 6 and 12 months in each of the intervention arms. To investigate associations between hunger, abstinence, early and late weight gain.

### Outcome Measures

#### Acceptability

Participant acceptability is measured qualitatively by semi-structured interviewing and quantitatively by response and attrition rates as described below:

• Semi-structured telephone interviews are conducted after the participant has completed or dropped out of the trial. Participants who are happy to do so will give their consent to this at the start of the trial. To help them remember their thoughts and feelings 'of the moment' they are given the questions they will be asked at the start of the trial, with space to jot down notes, during the trial. The number of participants being interviewed will continue until theoretical saturation of responses has been reached. We will purposively sample interviewees to encompass the full range of attrition characteristics, for example, those who completed the trial, and those who dropped out of quitting, dieting or both in each of the three trial arms. Interviews will be audio recorded and transcribed verbatim. Participants will be asked what they found helpful and unhelpful and their reasons for dropping.

• Rates of response to participant invitation letters and posters.

• Rates of recruitment at telephone screening, at first consultation.

• Rates of drop out before randomisation.

• Rates of drop out after randomisation in each treatment arm.

• Rates of attendance at each session.

• Rates of participant adherence to treatment.

• Quality of life measure, a scale on which the participant can score general life satisfaction and well being at baseline, weeks +4, and +12.

#### Feasibility

Feasibility of running the trial within the primary care practice will be measured qualitatively as described below:

• A focus group of participating clinicians to investigate experiences of delivering intervention, e.g. how easy was it to carry out the interventions, was training sufficient, was consultation time adequate, what difficulties were encountered taking the trial measurements.

• Principal investigator's reflections on their experiences of primary care involvement e.g. ease of GP practice recruitment, willingness for PCTs to participant, obstacles encountered.

#### Cost

The financial cost of running the trial will be calculated.

#### Measurement of urges to smoke and hunger

Degree of smoking addiction is measured at baseline using the Fagerstrom Score [31]. Urge to smoke is measured by the Mood & Physical Symptoms Combined Scale (MPSS-C) [32]. Hunger and food craving is measured by the Hunger and food Craving (HCS) score. MPSS-C and HCS are recorded daily in a dairy over the first four weeks of quitting and weekly thereafter. The primary outcomes of interest will be over the first 24 hours and the first week of quitting; this is where the largest effects are likely to be seen as withdrawal symptoms are at their peak during this time. Comparisons between measures are made for all those who continued in their quit attempt until they decided to abandon quitting, and adjusted for those who lapsed, were point prevalent abstinent at 24 hours, 7days or continuously abstinent at 4 weeks. Although it is standard practice in smoking trials to primarily analyse those who are abstinent, our interest is in the effects on cravings and we cannot assume that those who did not achieve abstinence did not experience cravings as they tried to do so. Piasecki showed that cravings were heightened in both smokers who were attempting to quit and lapsed as well as in smokers who succeeded in quitting. He found that more cigarettes, smoked on more occasions did reduced cravings (presumably as these have served to treat nicotine withdrawal acutely) but also that a few cigarettes increased cravings (100 vs 56 cigarettes p < 0.001) [33]. Perhaps this reflects a greater struggle to cope with cravings before a cigarette was finally smoked out of desperation.

#### Ketosis

The presence or absence of ketones will be measured using ketostik test strips dipped into urine samples. This will be done weekly during the first 6 weeks of dietary intervention, this is to check those in the VLCD are in ketosis throughout the five weeks and come out of ketosis during the re-feeding week. It is also measured during this time in IDAP and control arms to verify the absence of ketosis in these groups or identify any participants which might be self-imposing excessive dietary restriction, (unless undiagnosed diabetes presents itself).

#### Measurement of smoking status

Twenty-four hour point prevalence abstinence is measured by participants achieving 24 consecutive hours of abstinence as verified by exhaled CO < 10 ppm. Seven day point prevalence abstinence is defined as those not smoking over the last 7 days as verified by exhaled CO < 10 ppm. Participants achieving 1, 6 and 12 month abstinence as defined using the Russell Standard which states that no more than 5 cigarettes since have been smoked since week +2, this is verified by CO < 10 ppm at each consultation [34]. Participants who have not achieved abstinence but are still attempting to quit are termed 'lapsed'. Smokers who abandon their quit attempt, such that it is no longer their intention to quit, are considered 'relapsed'.

#### Measurement of Disease Risk Factors

These are measured as described below. The schedule of measurements is contained in table 2.

**Table 2:** Measurements to be taken at each point in trial

Treatment week	-2	-1	0	1	2	3	4	5-7	8-10		12	26	52
Baseline Questionnaire (with Fagerstrom Score) 7 day food diary		√											

Weight		√	√	√	√	√	√	√	√	√	√	√	√

Waist/Hip ratio		√	√	√	√	√	√	√	√	√	√	√	√

BP/HR		√	√	√	√	√	√	√	√	√	√	√	√

Smoking Status		√	√	√	√	√	√	√	√	√	√	√	√

Urinary ketones		√	√	√	√	√	√						

Fasting Blood test	√										√	√	√

Lung function	√										√	√	√

Russell Standard									√		√	√	√

QOL measure		√					√				√		

Confidence in trial arm	√						√				√		

Percentage Body Fat composition		√	√	√	√	√	√	√	√	√	√	√	√

Daily Diaries		√	√	√	√	√	√						

Weekly Diaries								√	√	√	√	√	√

HCI		√									√	√	√

1. Weekly weight, waist to hip ratio, % body fat composition, blood pressure, and heart rate.

2. FEV1 and FVC post 200 mg salbutamol as is recommended by the American Thoracic Society 2005 standards for measuring lung function [35] at baseline, 3, 6 and 12 months.

3. Fasting blood glucose, Total cholesterol (TC), low density lipoprotein (LDL) cholesterol, high density lipoprotein (HDL) cholesterol TC/HDL ratio, triglycerides, haemoglobin, white blood cell count, platelet count, mean cell volume and c-reactive protein (CRP).

Full details of how these measurements are taken are contained within the trial clinical protocol as standard operating procedures (SOPs) and work instructions (WIs). The nurses are trained in these procedures so that they are carried out consistently at each trial site. The nurses are assessed in practice against these SOPs and WIs, every couple of months, by the principal investigator.

#### Measurement of diet and activity

Food choices, eating behaviour and activity are measured at baseline, end of treatment, 6 and 12 months using the HCI. Agreement between this simple, quick measure of diet quality will be assessed by statistical analysis against a detailed seven day food intake diary which is also completed at baseline. Participants are encouraged to weigh their food when completing the seven day food diary. Despite under-reporting in obese individuals, a 7 day weighed intake diary has been shown to be the most accurate measure of dietary intake [36]. Diaries which report less than 1.2 × BMR energy intake or are incomplete are discounted. If necessary the Goldberg cut-off can be used to evaluate the mean population bias in reported energy intake [37]. The seven day diaries also measure physically activity levels using the method which determined dietary references values for energy in the UK.

Those on a VLCD who do not produce ketones or achieve the expected 2 kg weekly weight loss will be considered non adherent. We would expect any weight gain in the IDAP group to be a result of poor adherence, although weight maintenance is acceptable in this group.

#### Confidence in trial arm

Due to the unblinded nature of the trial, participants are asked prior to randomisation to rate their confidence of each treatment arm being successful. This is measured again at +4 and +12 weeks. We will be able to determine whether expectation of success in the treatment they were allocated is associated with attrition rates.

### Sample Size

Acceptability and feasibility is measured qualitatively through interviews and by measurement of recruitment and attendance rates. These outcomes are descriptive and not analysed using statistical tests and so a power calculation for them would be inappropriate.

The secondary aim is to identify whether dietary interventions affect cravings for cigarettes. In the trial by Danielsson [25] the difference in cravings for cigarettes at week 2 was mean 1.6 in the control and mean 1.1 in the VLCD group with a standard deviation of 0.7. The control in the Danielsson trial was a standard (not individualised) 1600 kcal diet, a moderate energy restriction, likely to lead to increased hunger. We are looking to detect a difference of the same magnitude between our hungry (IDAP) and not hungry (control) or hunger suppressed (VLCD) groups. Using Epicalc with 80% power and a type 1 error rate of 5% we need 30 participants in each group and 90 in total to detect a significant difference between them. For sufficient power to detect a difference in an abstinent subgroup, assuming a 60% abstinent rate in the first few weeks, we are aiming to recruit 42 in each group.

Such a trial would be large enough to differentiate between dietary changes that reflect a poor to a healthy diet using a dietary index, this is based on the figures by Freisling et al in 2009 [38] who validated a food frequency index using values of <32 and >39 for a poor and very good diet respectively. With a standard deviation of 5.7, 10 people would be needed in each arm to detect a difference.

Running a larger trial to identify long term effects on abstinence and weight is premature at this stage, although we are carrying out all the measurements that would be needed in such a trial to assess feasibility.

### Randomisation

Randomisation is computer generated by an independent statistician within the Primary Care Clinical Research and Trials Unit (PCCRTU) using random permuted blocks of length 6, stratified by practice. The numbers are entered into the trial database by an independent computer programmer within the trials unit. The database conceals randomisation until after participants have been screened and entered into the trial. At week -3 the clinician clicks on the randomisation tab in the database and this reveals the arm to which the participant is allocated. The database is set up so that the randomisation 'tab' will not work until all data from week -2 has been completed. Therefore it is impossible for anyone to see treatment allocation beforehand. This greatly minimises any risk of the trial randomisation being undermined.

### Analysis and Statistical Methods

Semi-structured interview and focus group transcripts will be coded according to content and common themes regarding acceptability and feasibility will be identified.

Quantitative measures of acceptability will be presented as descriptive statistics.

The outcomes between the three arms of the study will be compared using statistical tests on adequately powered measures. Descriptive statistics (mean, SD and 95% CI) will be presented on underpowered measures.

Multilevel modelling based on the Piasecki model of cigarette withdrawal [33] will be used to investigate the effects of the dietary interventions on urges to smoke during the first 4 weeks of quitting. We will investigate whether these effects are mediated by hunger/food craving score (HCS) and ketosis. We will adjust for confounding variables e.g. active treatment for depression. Analysis will be carried out on all those who continued to try to quit regardless of lapses to smoking and this will be adjusted for in the model as described above.

Significance is set at the 5% level, exact p values will be given and 95% confidence intervals where appropriate.

Analysis of data from treatment stage 1 will be undertaken once every participant has completed this stage. Analysis from treatment stage 2 and follow-up at 6 months and one year will be undertaken once participants have gone through each of these phases.

### Data Validation

Data cleaning will take place by a series of logical checks on the electronic data. (For example, a person cannot be recorded as prolonged abstinent smoker at 6 months if they were not in such a state at 8 weeks). Discrepant records are checked with the source documents and the database amended if necessary.

### Trial schedule

One Doctoral researcher is principal investigator and supervising dietitian. Part-time support is provided by practice research nurses and research administrators.

Recruiting will continue over a period of a year until sufficient participants have been treated in each trial arm. Follow up will take place as described and it is estimated that the trial will be complete 2 years from the start.

### Definition of end of trial

End of trial is defined as the final 12 month follow-up where the last measurement is taken from the last participant and the last participant undergoing the trial is debriefed.

### Value of Results

The results from this study will provide new information about dieting during smoking cessation. They will inform the design of a multi-component intervention that tackles both smoking cessation and its related weight gain in a way which can be rolled out into the NHS.

### Assessment of safety

Potential participants' safety is ensured by screening for eligibility using a structured form completed by the trial healthcare professionals. This will record evidence of eligibility and that the person does not have any exclusion criteria. In addition, the nurse will take a general medical and drug history to assess for other complicating diseases. Any queries remaining as a result of this process are resolved by discussion between the trial nurse, chief investigator and the relevant physicians providing routine medical care, usually the participant's GP. Such concerns are unusual but not rare. Typically, they arise from a participant's hazy knowledge or understanding of their past medical history and are usually readily resolved. No blood or further medical testing is necessary to ensure safety.

#### VLCDs

Very low calorie diets are a recognised treatment for obesity. They form part of the NICE (2006) guidelines [14] for the management of adult obesity and are advised for up to three months of continuous use in people with a BMI > 30. We are using it for 5 weeks only in people with a BMI > 25. Weekly monitoring of weight means that if BMI falls below a healthy level treatment will be discontinued. VLCDs have been used safely for many years including in people with a BMI between 25 and 30 [16]. Since 1987 they have been subject to the regulations of the Committee on Medical Aspects of Food [39]. This is an extensively researched evidence based document detailing the formulation of VLCDs to ensure safety. The product to be used in this study complies with these standards. Thus, there is every reason to expect that treatment in this trial is safe.

Participants are warned about the side-effects of VLCDs and may contact the trial team to discuss any concerns. To this end, all participants are given a card the size of a credit card with the trial team's contact details on that will allow participants to receive advice on the VLCD or to report perceived serious adverse effects and receive advice as required. They are asked to carry this card with them at all times so that it can be used to notify medical personnel of a participant's treatment and trial involvement in case of emergency. Participants will record the occurrence of side-effects of VLCDs as specified by completing a checklist. The checklist is given to the trial healthcare professional and the healthcare professional will enquire about recorded adverse events, so as to determine the severity of any adverse event and ensure that appropriate advice is given for its management (e.g. drinking appropriate amounts of water to treat symptoms of mild dehydration.) Minor adverse reactions are monitored and managed in this way. For each known side effect listed in the checklist, the trial healthcare professional will have a definition of clinical severity. Any side effect that is classified as moderate or severe is reported to and discussed with the principal investigator. A decision on stopping therapy will then be made with the participant, attending clinician, principal investigator and other relevant parties as appropriate.

#### Dietary and Lifestyle Advice

Healthy dietary and lifestyle advice is individually tailored to create a mild energy deficit and gentle increase in activity. This advice is given by appropriately trained healthcare professionals. It is usual practice and considered very safe. In the unlikely event of side effects participants may contact the trial team to discuss any concerns. To this end, all participants are given a credit card-sized card with the trial team's contact details on that will allow participants to report perceived serious adverse effects and receive advice as required.

#### NRT Patches

NRT has been investigated in several hundred previous clinical trials and is widely prescribed worldwide and subject to safety monitoring, and is replacing a product, nicotine, which the participants are already consuming and will have consumed for many years in cigarettes. Thus, there is every reason to expect that treatment in this trial is safe. Participants are warned about the side-effects of NRT and advised not to stop taking the medication without consulting with the trial team or an NHS professional if the trial team are unavailable. Participants will record the occurrence of side-effects of medication as specified on the summary of product characteristics (SPC) for all relevant NRT preparations, by completing a checklist. The checklist is given to the trial healthcare professional and the healthcare professional will enquire about recorded adverse events, so as to determine the severity of any adverse event and ensure that appropriate advice is given for its management (such as rotating the patch site or use of emollients for skin reactions). Minor adverse reactions are monitored and managed in this way. For each known side effect listed in the SPC, the trial healthcare professional will have a definition of clinical severity. For example, a mild skin site reaction to the patch is defined as burning sensation that does not interfere with normal activities, redness or swelling at the site of application, or mild blistering. Any reaction beyond that is classified as potentially moderate or severe and is reported to and discussed with the principal investigator. A decision on stopping therapy will then be made with the participant, attending clinician, principal investigator, and other relevant parties as appropriate. Nicotine has a short half life (2 hours), meaning that the blood concentration will not build up during the course of treatment so that new side-effects are not expected after the first few weeks. In addition, reactions to it relate to local use, such as skin discomfort from patches and people become accustomed to the side-effects after a short time of using the preparation. The advice given will depend upon the severity of the reported reaction and those with moderate reactions are invited to an ad hoc consultation.

The SPCs for the relevant NRT products contain no warnings about serious adverse reactions except rare allergic reactions, such as angioedema, and cardiac arrhythmias, occurring in less than 1/1000 users. Thus we expect no or very few SUSARs (suspected unexpected serious adverse reaction) in this trial. The long history of use in and outside of trials for NRT means that SUSARs are unlikely. On the reverse of the trial card given the contact number for advice on side-effect management, there are instructions for reporting of serious adverse events. Through direct contact from the participant or contact from their attending physician, we expect to become aware of serious adverse events. If any member of the trial team becomes aware, they will inform the principal investigator within 24 hours. The principal investigator will then assess the seriousness, causality, expectedness and severity of the adverse effects. An immediate decision is made on the interim use of medication for that participant. If an event is judged severe, it is reported to the trial sponsor, who will report the event to the REC. Definitions of adverse events are contained in additional file 3.

#### Salbutamol

Salbutamol has been thoroughly investigated in clinical trials and is widely prescribed worldwide and subject to safety monitoring. Thus, there is every reason to expect that its use in this trial is safe. Common side effects to salbutamol are mild (e.g. headache, tremor) and rare with small doses; severe reaction is very rare. Any reaction tends to be immediate. Participants are warned about the side-effects of salbutamol and asked to give verbal consent to taking it. They will take a small dose (200 mcg) in the company of healthcare professionals and given a contact number should they experience any adverse events in the hours that follow. This is administered 4 times during the year, at baseline, 12 weeks, 6months and 1 year. For each known side effect listed in the SPC, the trial healthcare professional will have a definition of clinical severity. Any reaction beyond that is classified as potentially moderate or severe and is reported to and discussed with the principal investigator. If it is felt that a person has suffered side-effects which are related to the salbutamol then they will not have the salbutamol administered next time.

The long history of use in and outside of trials for salbutamol means that SUSARs are unlikely. On the reverse of the trial card given the contact number for advice on side-effect management, there are instructions for the reporting of serious adverse events. Through direct contact from the participant or contact from their attending physician, we expect to become aware of serious adverse events. If any member of the trial team becomes aware, they will inform the principal investigator within 24 hours. The principal investigator will then assess the seriousness, causality, expectedness and severity of the adverse effects. If an event is judged severe, it is reported to the trial sponsor, who will report the event to the Research Ethics Committee (REC). Definitions of adverse events are contained in additional file 3.

Participants are asked weekly to report intercurrent illnesses and the response recorded. If any of these intercurrent illnesses contra-indicates Salbutamol, NRT, VLCD or Healthy Dietary Advice, this is immediately reported to the principal investigator and a decision made about continued use.

### Ethics and Research Governance

The trial is conducted in compliance with the principles of the Declaration of Helsinki (1996), the principles of the International Conference on Harmonisation (ICH)-Good Clinical Practice (GCP) and run in accord with EU Clinical Trials Directive and all of the applicable regulatory requirements. The study protocol and other documentation have been approved by South Birmingham Research Ethics Committee, Birmingham and Black Country Comprehensive Local Research Network and West Midlands South Comprehensive Local Research Network. Any subsequent protocol amendments will be submitted to the REC for approval and the other bodies if necessary. We will comply with ICH-GCP Guidelines over the reporting of adverse events (AEs), serious adverse events (SAEs) and suspected unexpected serious adverse reaction SUSARs. In addition we will provide the REC with progress reports as well as a copy of the final study report.

### Data management, protection and confidentiality

The trial is being run as part of the portfolio of trials in the Primary Care Clinical Research and Trials Unit (PCCRTU), NIHR accredited trials unit number 33, in Primary Care Clinical Sciences at the University of Birmingham. The data management is run in accord with the standard operating procedures (SOPs), which are fully compliant with the Data Protection Act and ICH GCP. The trial registered with the Data Protection Act website at the University of Birmingham. Patient identifiable data is shared only within the clinical team on a need-to-know basis to provide clinical care and ensure good and appropriate follow up. Patient identifiable data will also be shared with the GP and approved auditors from the REC or NHS Research & Development (R&D) will also be able to see patient identifiable information. Otherwise, confidentiality is maintained and no one outside the trial team will have access to either the case report forms (CRFs) or the database. The source documents for the trial are CRFs which are stored in a locked cabinet at the participating practice. The trial database is securely held and maintained by the PCCRTU. On completion of the trial and data checking, the CRFs are transferred to Modern Records, a secure archiving facility at the University of Birmingham, where they are held for 15 years and then destroyed. The database is anonymised and a secure CD containing the link between ID number and patient identifiable information is stored in modern records.

### Finance

The study is funded by UK Centre for Tobacco Control Studies (UKCTCS) and service support costs from the Comprehensive Clinical Research Network.

### Publication

The trial results will be written up for submission to a peer reviewed journal and the trial is registered with ISRCTN. No data relating to individuals will be identified in these publications.

## Competing interests

Deborah Lycett declares no competing interests. Paul Aveyard has done consultancy work on smoking cessation for Pfizer, McNeil, and Xenova Biotechnology. Peter Hajek received research funding and provided consultancies to manufacturers of stop-smoking medications.

## Authors' contributions

DL and PA designed the study and wrote the manuscript. PH provided advice on both. All authors have read and approved the manuscript.

## Supplementary Material

Additional file 1 **Substantial Amendment**. Details of an approved substantial amendment to this trial protocol regarding improving recruitment rates. Click here for file

Additional file 2 **Safe Use of Low Dose Diuretics with a VLCD**. Clinical protocol, for use in this trial only, for the safe use of a VLCD with low dose diuretics used to treat hypertension. Click here for file

Additional file 3 **Adverse Event Reporting**. Definitions of adverse events and responsibilities for reporting them. Click here for file
