# Supplementary material for: Trial Protocol: Randomised controlled trial of the effects of very low calorie diet, modest dietary restriction, and sequential behavioural programme on hunger, urges to smoke, abstinence and weight gain in overweight smokers stopping smoking
Source: Trials. 2010 Oct 7;11:94. doi: 10.1186/1745-6215-11-94 (PMC2959046; doi:10.1186/1745-6215-11-94)
Supplement: Additional file 1 — Substantial Amendment. Details of an approved substantial amendment to this trial protocol regarding improving recruitment rates. [file 1745-6215-11-94-S1.DOC]

**Additional file 1**

**Substantial Amendments to DeMiST Protocol May 2010**

**Background**

Recruitment rate from letters sent out from GP practices is currently 1%. We are only identifying a limited number of potentially eligible participants at individual practices such that we would we would need about 20 practices in total and each would only be giving us 5 participants. This would require a lot of investment for a very small return. The reason for these small numbers is two-fold, firstly our target population of overweight smokers is relatively small, secondly the extensive exclusion criteria, for taking a VLCD, is reducing our numbers further. We therefore propose the following amendments to the protocol.

**Amendments**

1. To advertise more widely so that we can recruit from several practices at a time. We plan to do this under the auspices of the PCT stop smoking services. We plan a leaflet drop, using a local newspaper, to houses within the postcodes surrounding a health centre within that PCT. The change to the protocol is a follows, with amended content in bold.

*Recruitment*

Overweight (BMI>25Kg/m^2^) smokers listed on the databases of participating general practices are invited to take part by a letter from their General Practitioner (GP). **An advert will also be circulated through the local newspaper inviting potential participants to contact the research team or visit their website for more information...**

1. In order to increase our chance of recruitment we propose to randomise those who cannot meet recruitment criteria due to contraindications to taking a VLCD into a parallel 2 armed trial (IDAP and control). We will combine the data from the IDAP and control arms in both trials in a meta-analysis which will increase the power and generalisability of our results.

This will enable us to test our hunger hypothesis even if we recruit insufficient numbers to test our ketone hypothesis. The change to the protocol is a follows, with amended content in bold.

*...Inclusion Criteria*

- Aged over 18 years
- Daily cigarette smoker with an exhaled CO of at least 10ppm at least 15 minutes after last smoking
- Willing to be randomised to any of the three arms and willing and able to comply with the intervention and all study procedures
- BMI of at least 25kg/m2 **[if BMI is less that 25 may be eligible for 2 armed trial so can continue to assess exclusion criteria]**

*Exclusion Criteria*

- Any of the absolute or relative contraindications to VLCD use. These include... **[if contraindicated may be eligible for 2 armed trial – continue screening]**

- Uncontrolled hypertension and type 2 diabetes treated with medication. Although these are not contraindications to the VLCD they are excluded as adjusting the medications in these conditions would require specialist advice beyond the scope of the research nurses in this trial (diet controlled type 2 diabetics may be included). **[this is an exclusion due to the nature of the VLCD may be eligible for 2 armed trial- continue screening]**
- Those on oral anticoagulants, digoxin, phenytoin and lithium are excluded due to likelihood of increased drug absorption if on a VLCD. Specialist advice beyond the scope of our research nurses is needed to reduce and monitor these medications. **[this is an exclusion due to the nature of the VLCD may be eligible for 2 armed trial- continue screening]**
- Those on diuretics for diuresis are excluded as a VLCD can potentiate diuresis and increase the risk of hypokalaemia. Those on low dose diuretics for treatment of hypertension are not excluded as the risk is of hypokalaemic complications on these are low [18, 19]. The effects of combining a VLCD with low dose thiazides has not been well studied and may be unpredictable therefore serum potassium will be monitored weekly and the VLCD discontinued if levels fall below 3.1mmol/l. (Appendix 1 contains full list of excluded medications). **[this is an exclusion due to the nature of the VLCD may be eligible for 2 armed trial- continue screening]**

*Randomisation*

Patients are randomised to one of the three study arms by computer generated random permuted blocks of length 6, stratified by practice. **For those who do not meet the exclusion criteria due to contraindications for VLCD use they will be randomised independently into either the IDAP or SBS arm.**

*Statistical Methods*

The analysis will compare the outcomes between the three arms of the study using statistical comparison tests on adequately powered measures. Descriptive statistics (mean, SD and 95% CI) will be presented on underpowered measures. **Results from the two way independent randomisation (IDAP and SBS) will be combined meta-analytically with the three-way independent randomisations.**

1. Where clinical facilities aren't available at community health centres we need the freedom to obtain an alternative measurement other than venous blood tests and. We have already found in our trial that arranging one practice to carry out venous blood tests for another is very difficult practically with the potential for measures to be missed and results lost. Although fasted venous samples may be biochemically superior we feel that the benefit of a simple system of capillary blood sampling using skin pricks will outweigh this in practice and provide adequate results in more people.

*Measurement of disease risk factors*

...Fasting blood glucose, Total cholesterol (TC), low density lipoprotein (LDL) cholesterol, high density lipoprotein (HDL) cholesterol TC/HDL ratio, triglycerides, haemoglobin, white blood cell count, platelet count, mean cell volume and c-reactive protein (CRP). **Where it is impractical to take such samples random blood glucose and cholesterol measures using a skin prick device will be taken.** P**articipant will be asked to make an appointment with their GP if the results show cause for concern.**
