# Supplementary material for: Trial Protocol: Randomised controlled trial of the effects of very low calorie diet, modest dietary restriction, and sequential behavioural programme on hunger, urges to smoke, abstinence and weight gain in overweight smokers stopping smoking
Source: Trials. 2010 Oct 7;11:94. doi: 10.1186/1745-6215-11-94 (PMC2959046; doi:10.1186/1745-6215-11-94)
Supplement: Additional file 2 — Safe Use of Low Dose Diuretics with a VLCD. Clinical protocol, for use in this trial only, for the safe use of a VLCD with low dose diuretics used to treat hypertension. [file 1745-6215-11-94-S2.DOC]

**Additional file 2**

**The safe use of low dose diuretics for treatment of hypertension when on a VLCD**

**Background**

Diuretics stimulate diuresis and are used to treat oedema and hypertension. This loss of body water increases potassium excretion and may lead to low serum potassium levels (hypokalaemia). Hypokalaemia is particularly dangerous in severe cardiovascular disease and hepatic failure. Serum potassium should be regularly monitored. Potassium sparing diuretics or potassium supplements are used to prevent and treat hypokalaemia (BNF, 2008).

A very low calorie diet causes diuresis as glycogen stores are metabolised to glucose and water. Glycogen stores are exhausted within the first week and so glycogen related diuresis occurs during this time (Lipotrim medical information, 2004).

The combination of a VLCD and diuretics increase diuresis and therefore increases the risk of hypokalaemia (Lipotrim medical information, 2004) but the extent is unclear.

When diuretics, most commonly the thiazides, are used in low doses for the treatment of hypertension, hypokalaemia which is considered mild (between 3.1 and 3.4mmol/l) occurs in the range of 7.2% and 50% of patients. (Franse et al 2000, Blanning et al, 2001, Peters et al, 1989). Studies have failed to show an increased cardiovascular risk associated with hypokalaemia from low dose thiazide use (Franse et al 2001, Peters et al 1989) and so we consider the risk of these to be low.

Large numbers of people are treated with low dose thiazides and excluding them would limit recruitment and the generalisability of our trial. We are therefore accepting those who are treated with diuretics for hypertension at the levels detailed in the appendix. Diuretics used for treatment for oedema may result in have greater potassium loss and so we consider these to be a higher risk. While potassium sparing diuretics used for treating oedema may minimise this risk they remain excluded as large shifts in body fluid will result in inaccurate body weight measurements. The excluded diuretics are also listed in the appendix.

Lipotrim contains high levels of potassium to take into account glycogen related diuresis, however the effects of a VLCD together with low dose thiazides has not been extensively studied and it impossible to predict outcome on an individual basis. The risk of falling potassium is greatest in the first week of a VLCD when glycogen stores are exhausted. However there is one case reported in the medical literature of a man (BMI 43.6kg/m2) on hydroclorathiazide, 20mmol KCl and a VLCD who had a serum potassium of 2.8mmol/l 15 days after initiating a VLCD (Liu et al, 2005). He had lost 13.2kg body weight during that time; which is greater than we would expect in our trial.

There is also some evidence from observational data that those who are obese and taking diuretics are more prone to hypokalaemia than their healthy weight counterparts (Mariosa et al, 2008).

For these reasons we will monitor serum potassium at baseline for anyone on a diuretic and continue to do so, on a weekly basis, for anyone who is on a diuretic and the VLCD formula. We will contact their GP and discontinue the VLCD according to the severity of hypokalaemia as detailed in the flow chart below. (Figure 1)

The flowchart is based on the clinical practice guideline by Smellie et al (2007) which classifies hypokalaemia and its management. Serum potassium levels between 3.3 and 3.5mmol/l are of little clinical relevance and unlikely to be treated. Levels between 3.0 and 3.3mmol/l are still mild but oral potassium replacement may be considered in some circumstances. Levels between 2.6 and 3.0mmol/l are considered moderately severe and oral treatment to replace potassium is usual. Levels below 2.6mmol/l are classed as severe and usually require hospitalisation for intravenous potassium replacement.

**Medication Exclusions for DeMiST**

**Stop smoking meds:**

NRT

Bupropion/Zyban

Varenicline/Champix

Nortriptyline

**Oral Anticoagulants:**

Warfarin

acenocoumarol (nicoumalone)

phenindione

Dabigatran etexilate

Rivaroxaban

**Anti-obesity med:**

Orlistat/Xenical

Sibutramine (no longer available)

**Anti-convulsive:**

Phenytoin

**Anti-depressant/mood stabiliser:**

Lithium

**Anti-arrhythmic:**

Digoxin

**Diuretics:**

Furosemide (Froop, Rusyde Frusol *Lasix)*

[*Bumetanide*](http://bnf.org/bnf/bnf/current/2356.htm) (Burinex)

Eplerenone (Inspra®)

Spironolactone (Aldactone)

Tria[mterene](http://bnf.org/bnf/bnf/current/2366.htm) (Dytac)

Co-Amilofruse (Frumil)

**Diuretics that can be included in DeMiST**

**The following CAN be included if doses are not above those stated:**

Bendroflumethiazide (Bendrofluazide Aprinox, Neo-NaClex Neo-NaClex-K) 2.5 mg Daily

Chlortalidone (Chlorthalidone) 25mg Daily

[Cyclopenthiazide](http://bnf.org/bnf/bnf/current/41001i529.htm) 250 micrograms Daily

[Indapamide](http://bnf.org/bnf/bnf/current/41001i536.htm) ([Natrilix®](http://bnf.org/bnf/bnf/current/2340.htm), [Ethibide Xl®](http://bnf.org/bnf/bnf/current/201655.htm)) 2.5mg Daily

Metolazone ([Metenix 5®](http://bnf.org/bnf/bnf/current/2346.htm)) 5mg Daily

Xipamide ([Diurexan®](http://bnf.org/bnf/bnf/current/2351.htm)) 20mg Daily

Amiloride **(**Amilamont®) 5mg Daily

Co-amilozide 2.5/25 **(**Moduret 25®) 1 tablet/day

Co-amilozide 5/50 (Amil-Co®, Moduretic®) ½ tablet/day

[Co-amilozide](http://bnf.org/bnf/bnf/current/2377.htm) (Navispare® ) 1 tablets/day

Co-triamterzide 50/25 (Triam-Co ® Dyazide) 1 tab/day

[Torasemide](http://bnf.org/bnf/bnf/current/128199.htm) (Torem®) 2.5mg daily

| **References**  Blanning A, Westfall JM, How soon should serum potassium levels be monitored for patients started on diuretics? Clinical Inquiries FROM THE FAMILY PRACTICE INQUIRIES NETWORK March 2001 (Vol. 50, No. 3)  British National Formulary 55, March 2008  Franse LV, Pahor M, Di Bari M, Somes GW, Cushman WC, Applegate WB. Hypokalemia associated with diuretic use and cardiovascular events in the systolic hypertension in the elderly program. Hypertension 2000;35:1025–30    Liu T. Nagami GT. Everett ML. Levine BS Very low calorie diets and hypokalaemia: the importance of ammonium excretion. Nephrology Dialysis Transplantation. 20(3):642-6, 2005 Mar.  Mariosa LS. Ribeiro-Filho FF. Batista MC. Hirota AH. Borges RL. Ribeiro AB. Zanella MT. Abdominal obesity is associated with potassium depletion and changes in glucose homeostasis during diuretic therapy."The journal of clinical hypertension 10.6 (2008):443-9.  Medical Information for the use of Lipotrim, Howard Foundation Research Limited January 2004  Peters RW, Hamilton J, Hamilton BP. Incidence of cardiac arrhythmias associated with mild hypokalemia induced by low-dose diuretic therapy for hypertension. South Med J 1989;82:966–69.  Smellie,W.S.A.; Shaw,N.; Bowlees,R.; Taylor,A.; Howell-Jones,R.; McNulty,C.A.M. Best practice in primary care pathology: review 9. Journal of Clinical Pathology 2007 60(9) 966-974 |
| --- |
