# Supplementary material for: Trial Protocol: Randomised controlled trial of the effects of very low calorie diet, modest dietary restriction, and sequential behavioural programme on hunger, urges to smoke, abstinence and weight gain in overweight smokers stopping smoking
Source: Trials. 2010 Oct 7;11:94. doi: 10.1186/1745-6215-11-94 (PMC2959046; doi:10.1186/1745-6215-11-94)
Supplement: Additional file 3 — Adverse Event Reporting. Definitions of adverse events and responsibilities for reporting them. [file 1745-6215-11-94-S3.DOC]

**Additional file 3**

**Adverse Event Reporting**

## Definitions

## Adverse event

An AE is any untoward medical occurrence in a clinical investigation subject administered a product or medical device; the event need not necessarily have a causal relationship with the treatment or usage. Examples of AEs include but are not limited to:

- abnormal test findings,
- clinically significant symptoms and signs,
- changes in physical examination findings,
- hypersensitivity, and
- progression/worsening of underlying disease.

Additionally, they may include the signs or symptoms resulting from:

- product overdose,
- product withdrawal,
- product misuse,
- drug interactions,

Failure of expected pharmacological action or therapeutic benefit alone (i.e. lack of efficacy) is not necessarily an AE.

Definition of serious adverse event

A serious adverse event or serious adverse drug reaction is any untoward medical occurrence at any dose that: results in death, is life-threatening (immediate risk of death), requires inpatient hospitalisation or prolongation of existing hospitalisation, results in persistent or significant disability/incapacity, and/or results in congenital anomaly/birth defect. An important medical event may not be immediately life-threatening and/or result in death or hospitalisation. However, if it is determined that the event may jeopardize the subject and may require intervention to prevent one of the other outcomes listed in the definition above, the important medical event should be reported as serious. Examples of such events are intensive treatment in an emergency room or at home for allergic bronchospasm; blood dyscrasias or convulsions that do not result in hospitalisation; or development of drug dependency or drug abuse.

### Definition of adverse reaction (AR)

Adverse reaction refers to any untoward and unintended response in a subject to an investigational product.

### Definition of suspected serious adverse reaction (SSAR)

Means an adverse reaction that is classed as serious and which is consistent with the information about the medicinal product in question set out

1. in the case of a licensed product, in the summary of product characteristics (SmPC) for that product, or
2. in the case of any other investigational medicinal product, in the Investigator’s Brochure (IB) relating to the trial in question

### Definition of suspected unexpected serious adverse reaction (SUSAR)

Means an adverse reaction that is classed as serious and which is not consistent with the information about the medicinal product in question set out

1. in the case of a licensed product, in the summary of product characteristics (SmPC) for that product
2. in the case of any other investigational medicinal product, in the Investigator’s Brochure relating to the trial in question

Monitoring and reporting adverse events

All observed or volunteered adverse events regardless of treatment group or suspected causal relationship to any of the dietary or nicotine replacement therapies will be reported as described in the following sections. For all adverse events, the investigator will pursue and obtain information adequate both to determine the outcome of the adverse event and to assess whether it meets the criteria for classification as a serious adverse event requiring immediate notification to the sponsor, the NHS R&D office, and the research ethics committee. The investigator will assess causality. For adverse events follow-up by the investigator is required until the event or its sequela resolve or stabilise.

Severity Assessment

If required on the adverse event case report forms, the investigator will use the adjectives mild, moderate, or severe to describe the maximum intensity of the adverse event. For purposes of consistency, these intensity grades are defined as follows:

- Mild- Does not interfere with subject’s usual function.
- Moderate- Interferes to some extent with subject’s usual function.
- Severe- Interferes significantly with subject’s usual function.

Note the distinction between the severity and the seriousness of an adverse event. A severe event is not necessarily a serious event. For example, a headache may be severe (interferes significantly with subject’s usual function) but would not be classified as serious unless it met one of the criteria for serious adverse events, listed above.

Causality Assessment

The investigator’s assessment of causality must be provided for all adverse events (serious and non-serious). An investigator’s causality assessment is the determination of whether there exists a reasonable possibility that the investigational product caused or contributed to an adverse event. If the investigator’s final determination of causality is unknown and the investigator does not know whether or not investigational product caused the event, then the event will be handled as “related to investigational product” for reporting purposes. If the investigator’s causality assessment is “unknown but not related to investigational product”, this should be clearly documented on trial records. In addition, if the investigator determines a serious adverse event is associated with trial procedures, the investigator must record this causal relationship, as appropriate, and report such an assessment in accordance with the serious adverse event reporting requirements, if applicable.

#### The CI/PI’s responsibilities and processes for evaluating AEs

Each AE will be evaluated for seriousness, causality, expectedness and severity. The responsibility for this will lie with Dr Paul Aveyard and then reported to the Sponsor. The flowcharts will show the study team how to assess AEs and SAEs and decide whether any event requires further expedited reporting by the Sponsor.

#### The CI/PI’s responsibilities, definitions and criteria for the evaluation of SAEs

If the AE is assessed as serious Dr Aveyard will report the event to the sponsor immediately or within 24 hours of being made aware of the event. An initial verbal report can be made but will be followed promptly with a detailed written report on the trial SAE form.

#### Evaluation of AEs for Causality

- Not Related. Onset of the event as relative to administration of the product, is not reasonable; or, another cause itself can explain the occurrence of the event
- Unlikely to be related. Onset of the event as relative to administration of the product, is possible but another cause itself can explain the occurrence of the event or there is no reasonable grounds for suspecting that the product could have caused the event.
- Possibly related. Onset of the event as relative to administration of the product is reasonable; however the event could have been due to another, equally likely, cause
- Probably related. Onset of the event as relative to administration of the product is reasonable and is more likely explained by the drug than by any other cause.
- Definitely related. Onset of the event as relative to administration of the product is reasonable and there is no other cause to explain the event; or a re-challenge (if feasible) is positive.

Recording of AEs

Dr Aveyard will record all AEs on to the appropriate Trial Recording Form and copies filed in the subject’s notes.

#### Sponsor’s responsibilities for AE recording and reporting

The sponsor will obtain all AE records and perform an evaluation with respect to *seriousness, causality and expectedness*. Expedited reporting will be required where the AE has a possible causal relationship to the trial intervention, and/or is unexpected.

#### What the Sponsor will do following receipt of SAE report from CI/PI

On receipt of each and every SAE form the sponsor will provide an evaluation of ‘expectedness’.

All SAEs related to the medication that are both unexpected and serious, are subjected to expedited reporting. Other safety issues also qualify for expedited reporting, where they might alter the current risk-benefit assessment of the IMP; or where the issue may be sufficient to consider changes in the IMP administration or overall conduct of the trial, i.e. new events that relate to the conduct of the trial or the development of the IMP likely to affect the safety of participants ie: lack of efficacy of an IMP in the treatment of a life threatening disease single case reporting of an expected SAE, but with an unexpected outcome an increase in the rate of occurrence or severity of an expected SAE, judged to be clinically important post study SUSARs that occur after the subject has completed a trial.

Timeframes in which the Sponsor will submit expedited reports to the Research Ethics Committee (REC)

Fatal/life threatening SUSARs

The sponsor will inform the REC of the above as soon as possible, but no later than 7 calendar days after he has first knowledge of the minimum criteria for expedited reporting.

Non-fatal and non-life threatening SUSARs

The sponsor will report all other SUSARs and safety issues to OXREC as soon as possible but no later than 15 calendar days after he has first knowledge of the minimum criteria for expedited reporting.

Reporting other safety issues

A letter entitled Safety Report will be sent to the REC where other safety issues also qualify for expedited reporting by the sponsor. The first page will contain the title of the trial and the trial protocol code number.

The Co-ordinator of the main REC will acknowledge receipt of safety reports within 30 days.
